# Effects of Dipeptidyl Peptidase-4 Inhibition with MK-0431 on Syngeneic Mouse Islet Transplantation

**DOI:** 10.1155/2014/795283

**Published:** 2014-08-04

**Authors:** Jyuhn-Huarng Juang, Chien-Hung Kuo, Ying-Hsiu Liu, Han-Ying Chang, Chiung-Tong Chen

**Affiliations:** ^1^Division of Endocrinology and Metabolism, Department of Internal Medicine, Chang Gung University and Chang Gung Memorial Hospital, 5 Fu-Shin Street, Kweishan, Taoyuan 33305, Taiwan; ^2^Biomedical Technology and Device Research Laboratories, Industrial Technology Research Institute of Taiwan, 195 Section 4, Chung Hsing Road, Chutung, Hsinchu 31040, Taiwan; ^3^Institute of Biotechnology and Pharmaceutical Research, National Health Research Institutes, 35 Keyan Road, Zhunan, Miaoli 35053, Taiwan

## Abstract

Dipeptidyl peptidase (DPP)-4 inhibitors increase circulating levels of glucagon-like peptide-1 and glucose-dependent insulinotropic polypeptide which may promote *β*-cell proliferation and survival. This study tested if DPP-4 inhibition with MK-0431 is beneficial for diabetic mice syngeneically transplanted with a marginal number of islets. We syngeneically transplanted 150 C57BL/6 mouse islets under the kidney capsule of each streptozotocin-diabetic mouse and then treated recipients with (*n* = 21) or without (*n* = 17) MK-0431 (30 mg/kg/day, po) for 6 weeks. After islet transplantation, blood glucose levels decreased in both MK-0431-treated and control groups. However, the blood glucose and area under the curve of the intraperitoneal glucose tolerance test at 2, 4, and 6 weeks were not significantly different between MK-0431-treated mice and controls. During 6 weeks, both groups exhibited increased body weights over time. However, the weight between two groups did not differ throughout the study period. At 6 weeks after transplantation, the graft beta-cell mass (0.024 ± 0.005 versus 0.023 ± 0.007 mg, *P* = 0.8793) and insulin content (140 ± 48 versus 231 ± 63 ng, *P* = 0.2939) were comparable in the MK-0431-treated group and controls. Our results indicate posttransplant DPP-4 inhibition with MK-0431 in the diabetic recipient with a marginal number of islets is not beneficial to transplantation outcome or islet grafts.

## 1. Introduction

Recently, human islet transplantation has achieved insulin independence in type 1 diabetes and the success rates have been markedly improved [1]. However, most successful cases need 2 or more implants and the long-term follow-up shows their insulin independence declines with time [2, 3]. Therefore, the critical issue in clinical islet transplantation is to further improve and maintain its successful rate. Allograft failure may be due to nonimmunological (e.g., insufficient beta-cell mass and islet engraftment problems) as well as immunological (e.g., immune rejection, toxicity of immunosuppressants, and autoimmune recurrence) factors. To improve the outcome of islet transplantation, these problems have been intensively investigated [4]. The shortage of human donor pancreata has prompted efforts to expand the human donor pool and modify islet processing and preservation methods as well as identifying alternative islet sources. Another important approach is the generation of new beta-cells either from preexisting beta-cells or from progenitor/stem cells.

The glucagon-like peptide (GLP)-1 improves glycemic control in type 2 diabetic patients by stimulating glucose-dependent insulin secretion and biosynthesis and by suppressing glucagon secretion, gastric emptying, and appetite [5, 6]. Additionally, GLP-1 is also known to expand beta-cell mass by stimulating *β*-cell proliferation and inhibiting *β*-cell apoptosis [7–10]. However, clinical application of native GLP-1 is limited due to its very short plasma half-life [11]. Exenatide (exendin-4) is a GLP-1 receptor agonist resistant to dipeptidyl peptidase (DPP)-4-mediated inactivation and thus exhibits more sustainable effects [12]. It also had the ability to expand beta-cell mass via stimulation of beta-cell replication and neogenesis as well as prevention of beta-cell death in rodents [4]. We and others have shown exendin-4 not only improved transplantation outcome [13–15] but also expanded the graft beta-cell mass [15]. In contrast, DPP-4 inhibitors increase circulating active incretin hormones, GLP-1, and glucose-dependent insulinotropic polypeptide (GIP), by blocking their degradation [11]; thus, they can be beneficial for beta-cells. Previously, many studies have shown DPP-4 inhibitors improved glucose tolerance, insulin secretion, beta-cell glucose responsiveness [16], and insulin sensitivity [16, 17]; promoted beta-cell survival [18–20], islet neogenesis [18, 21], and proliferation [22]; reduced beta-cell death [23]; and preserved beta-cell mass and function [24–26] in diabetic rodents. In contrast, there is limited information regarding the effects of DPP-4 inhibitors on islet transplantation. One report used positron emission tomography (PET) imaging and demonstrated DPP-4 inhibitor, MK-0431, protected against the loss of islet grafts in streptozotocin (STZ)-diabetic mice [20]. In that study, 300 islets transfected with rAD-TK were transplanted and PET imaging instead of histology was used to assess the graft islet mass. The aim of this study is to test if DPP-4 inhibition with MK-0431 could be beneficial to transplantation outcome and islet grafts in diabetic recipients with a marginal number of islets (150 islets). Here, we used freshly isolated islets for transplantation and directly measured graft beta-cell mass by immunohistochemistry with point counting morphometry and insulin content.

## 2. Materials and Methods

### 2.1. Animals

Male inbred C57BL/6 mice (National Laboratory Animal Center, Taipei, Taiwan), aged 8–12 weeks, were used as transplantation donors and recipients. The recipients were made diabetic by a single intraperitoneal injection of STZ (Sigma Immunochemicals, St. Louis, MO, USA, 200 mg/kg body weight, freshly dissolved in citrate buffer, pH 4.5). Before transplantation, diabetes was confirmed by the presence of hyperglycemia, weight loss, and polyuria. Only those mice with blood glucose above 350 mg/dL at 2 weeks after STZ injection underwent transplant. Blood glucose values were determined on blood obtained from the snipped tail, with measurements performed with a portable glucose analyzer (One Touch II, Lifescan Inc., Milpitas, CA, USA). The animal experiments were approved by the Ethics Committee of Chang Gung Memorial Hospital [27–29].

### 2.2. Islet Isolation

Under anesthesia with sodium amobarbital, pancreases were distended with 2.5 mL of RPMI-1640 medium (GIBCO BRL, Grand Island, NY, USA) containing 1.5 mg/mL of collagenase (collagenase from* Clostridium histolyticum*, type XI, Sigma Immunochemicals), excised, and incubated in a water bath at 37°C. Islets were separated by a density gradient (Histopaque-1077; Sigma Immunochemicals), and purified islets were then handpicked under a dissecting microscope. Islets >75 and <250 *μ*m in diameter were collected and carefully counted into groups of 150 islets [27–29].

### 2.3. Islet Transplantation

One hundred and fifty C57BL/6 mouse islets were syngeneically transplanted under left kidney capsule of each inbred STZ-diabetic mouse on the same day as the isolation. Blood glucose and body weight were measured periodically after transplantation and normoglycemia was defined as nonfasting blood glucose levels <200 mg/dL [27–29].

### 2.4. MK-0431 Treatment

After islet transplantation, twenty-one recipients were treated with MK-0431, 30 mg/kg/day po, for 6 weeks. Seventeen recipients who had not received MK-0431 served as controls.

### 2.5. Intraperitoneal Glucose Tolerance Test (IPGTT)

After an overnight fast, a 5% glucose solution (1.5 g/kg) was injected intraperitoneally, and blood glucose was measured at 0, 30, 60, 90, and 120 min by tail snipping. The IPGTT was performed at 2, 4, and 6 weeks after transplantation [27–29].

### 2.6. Removal of the Islet Graft

Six weeks after transplantation, animals intended for graft removal were anesthetized with amobarbital. An abdominal incision was made and the kidney was exposed. Under dissecting microscope, the kidney capsule surrounding the graft was excised and removed with the adherent graft. The weight of each graft was determined on a Mettler balance type AE200 (Mettler Instruments Corp., NJ, USA) [29].

### 2.7. Immunohistochemistry and *β*-Cell Mass of the Islet Graft

The removed grafts were fixed in formalin solution and processed for paraffin embedding and sectioning. Sections of grafts were stained for the endocrine *β*-cells with immunoperoxidase by a guinea pig anti-swine insulin antibody (Dako Co., Glostrup, Denmark). Graft *β*-cell mass was measured by point counting morphometry on immunoperoxidase stained sections. Each section was covered systematically using a 48-point grid to obtain the number of intercepts over *β*-cells, endocrine non-*β*-cells, and other tissues. The *β*-cell relative volume was calculated by dividing the intercepts over *β*-cells by intercepts over total tissue; *β*-cell mass was then estimated by multiplying *β*-cell relative volume by graft weight [28, 29].

### 2.8. Insulin Content of the Islet Graft

At 6 weeks after transplantation, the graft-bearing kidneys were removed and homogenized in acid ethanol. After homogenization, the samples were extracted overnight at 4°C. On the following day, they were centrifuged at 2,400 rpm for 30 min and the supernatant was stored at −20°C. The pellet was again homogenized in acid ethanol and insulin was extracted overnight. After centrifugation, this second supernatant was added to the first extraction sample. Insulin was measured by radioimmunoassay with rat insulin RIA kit (Millipore Corporation, Billerica, MA, USA) [27–29].

### 2.9. Statistical Analysis

Results were expressed as mean and standard error of the mean (M ± SEM). Paired and unpaired Student's *t*-test were employed to compare values in a group and values between two groups, respectively. A value of *P* < .05 was considered significant.

## 3. Results

### 3.1. Effects of MK-0431 on Recipients' Blood Glucose after Islet Transplantation

**Figure 1:**
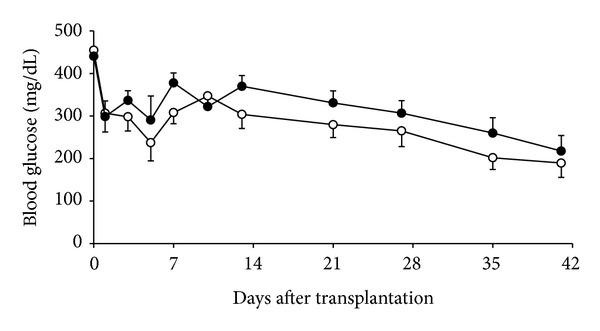
Blood glucose changes in islet recipients with (solid circle) and without (open circle) MK-0431 treatment.

After islet transplantation, recipients' blood glucose levels decreased progressively in both MK-0431-treated and control groups (Figure 1). However, the blood glucose levels were not significantly different between MK-0431-treated mice and controls throughout the study period. At 6 weeks, the blood glucose was 218 ± 37 and 189 ± 34 mg/dL in the MK-0431-treated group and controls, respectively (*P* = 0.5776).

### 3.2. Effects of MK-0431 on Recipients' Body Weight after Islet Transplantation

**Figure 2:**
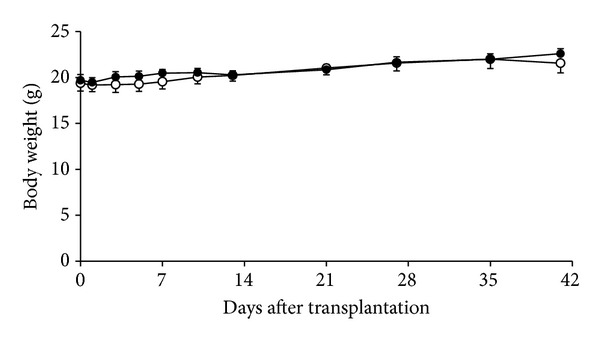
Body weight changes in islet recipients with (solid circle) and without (open circle) MK-0431 treatment.

During 6 weeks after islet transplantation, both groups exhibited increased body weights over time (MK-0431-treated group: 19.6 ± 0.7 to 22.6 ± 0.7 g, *P* = 0.0001; controls: 19.7 ± 0.9 to 21.8 ± 1.2 g, *P* = 0.0454) (Figure 2). However, the weight between two groups did not differ throughout the study period.

### 3.3. Effects of MK-0431 on Recipients' Glucose Tolerance after Islet Transplantation

**Figure 3:**
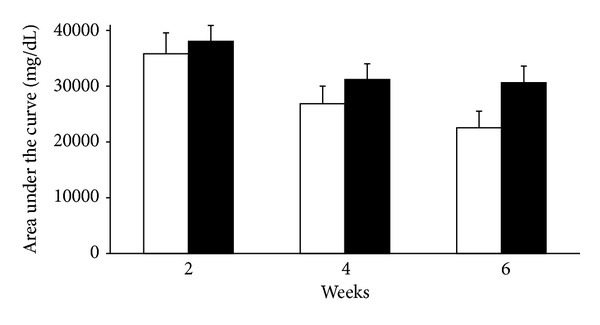
The area under the curve (AUC) of the intraperitoneal glucose tolerance test (IPGTT) at 2, 4, and 6 weeks in islet recipients with (black column) and without (white column) MK-0431 treatment.

After islet transplantation, the area under the curve (AUC) of the IPGTT at 2 weeks (38038 ± 2847 versus 35806 ± 3433 mg/dL, *P* = 0.6204), 4 weeks (31187 ± 2835 versus 26848 ± 3159 mg/dL, *P* = 0.3144), and 6 weeks (30634 ± 2954 mg/dL versus 22549 ± 2949, *P* = 0.0614) was not significantly different between MK-0431-treated mice and controls (Figure 3).

### 3.4. Effects of MK-0431 on Recipients' Graft Insulin Content and *β*-Cell Mass

**Figure 4:**
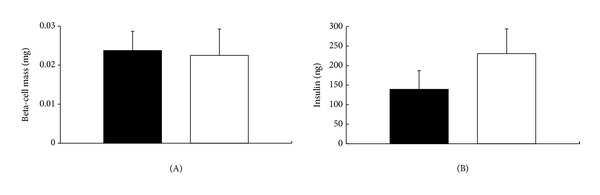
The graft *β*-cell mass (A) and insulin content (B) at 6 weeks in islet recipients with (black column) and without (white column) MK-0431 treatment.

At 6 weeks after transplantation, the graft beta-cell mass (MK-0431: 0.024 ± 0.005 mg, *n* = 14 versus controls: 0.023 ± 0.007 mg, *n* = 10, *P* = 0.8793) (Figure 4(A)) and insulin content (MK-0431: 140 ± 48 ng, *n* = 4 versus controls: 231 ± 63 ng, *n* = 5, *P* = 0.2939) (Figure 4(B)) were comparable in both groups.

## 4. Discussion

Although we and others previously showed exendin-4 improved transplantation outcome and expanded the graft beta-cell mass in diabetic mice transplanted with a marginal number of islets (150 islets) [15], this study demonstrated DPP-4 inhibition with MK-0431 did not. This observation is consistent with the fact that exendin-4 rather than DPP-4 inhibitors reduced blood glucose and increased pancreatic beta-cell mass in STZ-diabetic mice [19]. The above different effects on beta-cells are possibly due to the pharmacologic (exendin-4) and physiologic (GLP-1) bindings to GLP-1 receptors on beta-cells. In contrast, Lamont and Drucker treated high fat-fed mice with a remarkably higher dose (2.75 times) of MK-0431 which produced ~90% inhibition of plasma DPP-4 activity and found a significant reduction in glycated hemoglobin observed with DPP-4 inhibition but not with exendin-4 therapy. In addition, neither of the therapies increased beta-cell mass [30]. Although the effects of MK-0431 depend on its dose, we were unable to obtain sufficient plasma from mice to measure DPP-4 enzyme activity or active GLP-1 concentration after treatment with MK-0431. Hence, we cannot exclude the possibility that higher dose of MK-0431 may have beneficial effects on islet transplantation.

Our study design is different from that of Kim et al. who used PET imaging and showed MK-0431 protected against the loss of islet grafts in STZ-diabetic mice [20]. In contrast to our transplant with 150 freshly isolated islets, they transplanted 300 islets transfected with rAD-TK to each STZ-diabetic mouse. Our results are similar to their pilot studies in which mice receiving transplants of 100 islets remained hyperglycemic and did not benefit from treatment with MK-0431. Moreover, they assessed the graft islet mass by PET imaging which is not specific for beta-cells because [^18^F]FHBG was taken up by islets instead of beta-cells. In contrast, we directly measured graft beta-cell mass by point counting morphometry and insulin content which are more accurate than PET imaging for quantifying beta-cell mass. Even though we found MK-0431 is not beneficial to the beta-cell mass of 150-islet grafts, whether it did so to 300-islet grafts needs to be further confirmed.

## 5. Conclusions

Our results indicate posttransplant DPP-4 inhibition with MK-0431 in the diabetic recipient with a marginal number of islets is not beneficial to transplantation outcome or islet grafts.
